# Calcium carbide and gibberellic acid co-application enhances drought resilience in papaya (*Carica papaya* L.) by modulating photosynthetic efficiency and stress markers

**DOI:** 10.1186/s12870-025-07845-4

**Published:** 2026-01-16

**Authors:** Ili Nasleffa Rozman, Tinessha Paramasivam, Mohd Norsazwan Ghazali, Nur Indah Abdul Shukor, Khairul Azree Rosli, Md Aiman Takrim Zakaria

**Affiliations:** 1 Department of Crop Science, Faculty of Agriculture, Universiti Putra Malaysia, Serdang, Selangor 43400 UPM Malaysia; 2 Institute of Tropical Agriculture and Food Security (ITAFoS), Universiti Putra Malaysia, Serdang, Selangor 43400 UPM Malaysia

**Keywords:** *Carica papaya* L., Drought resilience, Calcium carbide, Gibberellic acid, Photosynthetic efficiency, Plant growth regulators

## Abstract

**Background:**

Papaya (*Carica papaya* L.), a critical tropical export crop generating 14 million tons yearly, shows extreme drought vulnerability due to shallow roots, high transpiration rate, and 85% tissue water content, exceeding other tropical fruits’ sensitivity. While individual calcium carbide (CaC_2_) and gibberellic acid (GA_3_) application show promise, their synergistic potential remains unexplored. We hypothesized that the dual Ca^2+^/acetylene release by CaC_2_ interact with the growth pathways of GA_3_ to create novel drought tolerance mechanisms.

**Results:**

Papaya seedlings were subjected to three water levels (100%, 75%, 50% field capacity) with four biweekly treatments over 12 weeks: control (water only), CaC_2_ (0.31 g plant^− 1^ surface-broadcast), GA_3_ (100 mg L^− 1^, 50 mL plant^− 1^ soil-drench), and CaC_2_ + GA_3_ combination. Co-application preserved photosynthesis under severe drought (27.48 vs. 7.78 µmol CO_2_ m^− 2^ s^− 1^ in controls) and maintained 78% optimal biomass. Principal component analysis revealed orthogonal relationships between stress markers and performance traits where treated plants reduced proline accumulation while maintaining growth, suggesting alternative osmotic adjustment pathways. Notably, nonstomatal limitations stayed below 1000 versus 2165 in controls, indicating preserved metabolic function. Even under well-watered conditions, combined application enhanced chlorophyll a by 75% and photosynthesis by 54%, demonstrating growth promotion beyond stress mitigation.

**Conclusions:**

The hormone-mediated physiological reprogramming via CaC_2_ and GA_3_ co-application suggests alternative drought tolerance response that decouple stress perception from growth suppression. This approach provides directly deployable technology for climate-resilient tropical agriculture, with important implications extending beyond papaya plants to other high-value tropical fruits facing intensifying climate extremes.

**Supplementary Information:**

The online version contains supplementary material available at 10.1186/s12870-025-07845-4.

## Introduction

Papaya (*Carica papaya* L.) is one of the most economically important tropical fruits globally, with Malaysia alone producing 55,974 metric tonnes across 2,412 hectares as of 2024 [1, 2]. As a high-value export crop, papaya cultivation contributes noticeably to agricultural revenue and rural livelihoods throughout tropical regions [3]. However, the increasing frequency and severity of drought events linked to climate change pose unprecedented challenges to sustainable papaya production practices [4–6]. Papaya is sensitive to soil water deficits in the early-seedling stage and even in adult plants [7]. These hydrological perturbations interrupt key physiological processes in papaya, compromising turgor pressure, nutrient uptake, and whole plant vigor, which ultimately causes significant yield reductions [8, 9].

While papaya’s optimal growth requirements are well-established, thriving at 25 °C with relative humidity ranging 60–85% [10], its adaptive responses to water deficit conditions remain inadequately understood. Recent research has shown that papaya employs complex stress signaling networks to regulate stomatal closure during drought periods, however these protective mechanisms often result in compromised photosynthetic efficiency and growth [11, 12]. This physiological trade-off highlights the urgent need for new approaches to enhance drought resilience in commercial papaya cultivation systems.

Plant growth regulators (PGRs) have arose as promising candidates for mitigating abiotic stress effects across various crop species [13, 14]. Gibberellic acid (GA_3_) is a multifunctional phytohormone that modulates various growth and developmental processes even under suboptimal conditions [15, 16]. Previous molecular and physiological studies have established the role of GA_3_ in upregulating hydrolase production, membrane integrity maintenance, and stress-responsive gene expression [17–19]. Concurrently, calcium (Ca) serves as a versatile secondary messenger that integrates environmental signals with cellular responses, markedly during stress episodes [20–22].

Despite extensive research on individual applications of either GA_3_ or calcium-based compounds in various crops [23, 24], limited information is available regarding their potential synergistic effects, particularly in drought-stressed tropical fruit species. Calcium carbide (CaC_2_) slowly releases Ca ions and acetylene gas in soil environments [25, 26], representing an underexplored agronomic input for stress alleviation [27, 28]. While acetylene gas can in theory be converted to ethylene through microbial activity [29], the exact mechanisms and extent of this conversion in soil remain to be fully characterized. However, both Ca signaling and potential ethylene release could interact with GA_3_, likely creating cross-talk mechanisms that may enhance stress tolerance more effectively than single-compound applications [30, 31].

This research introduces an innovative approach by studying the co-application of CaC_2_ and GA_3_ on papaya under systematically controlled water deficit conditions, a subject seldom investigated in tropical fruit cultivation systems. By examining the interaction effects of these compounds on crucial physiological and morpho-developmental traits across different field capacity levels, we address fundamental questions concerning optimal stress mitigation strategies. Unlike previous research focusing predominantly on vegetative growth metrics, our approach combines advanced physiological assessments, including photosynthetic parameters, chlorophyll responses, proline accumulation patterns, and multivariate statistical analyses providing mechanistic insights into drought adaptation processes.

The findings from this study hold important implications for developing climate-resilient papaya cultivation practices. By revealing the physio-biochemical basis of CaC_2_ and GA_3_ synergism under water stress, this research establishes a foundation for precision agriculture approaches that can be calibrated to specific drought intensity thresholds. This work also contributes to the emerging paradigm of hormone-mediated stress mitigation in tropical fruit production systems, providing theoretical and practical applications for sustainable agriculture in changing climatic conditions.

## Materials and methods

### Plant material and growth conditions

*Carica papaya* L. cv. “Sekaki” were received from a local trader (Rasa Pitaya Enterprise, Selangor, Malaysia). Three-week-old seedlings were transplanted into individual black poly-pots (40 cm x 40 cm) containing 5 kg of “Munchong” soil (Suppl. Table S1) under rain-shelter conditions with natural variations of light, temperature and relative humidity. Temperature and relative humidity oscillated around 60–99% and 24–50 °C, respectively. Throughout the experimental period, each plant was fertilized with 5 g of NPK blue 16: 16: 16 (YaraMila, Norway) every two weeks.

### Experimental design and treatments

A factorial experiment was carried out in a randomized complete block design with four biological replicates, from June to August 2024. The factors were three water stress levels: well-watered, 100% field capacity (WS100); mild, 75% field capacity (WS75); severe, 50% field capacity (WS50) and four growth enhancer treatments: control, no growth enhancer (GE1); CaC_2_ (GE2); GA_3_ (GE3); CaC_2_ + GA_3_ (GE4). The water stress and growth enhancer treatments were each initiated at 0 and 7 days after transplanting (DAT). Field capacity was determined using gravimetric method [32]. The measured gravimetric water content was 29.8%, indicating the mass of water relative to the dry soil mass. Therefore, the water volume based on field capacity rates of 100, 75, and 50% were 745, 1117, and 1490 mL per poly-pot, respectively. The target pot weights for maintaining field capacity treatments were established at the beginning of the experiment by saturating soil and allowing drainage for 24 h. Poly-pots were weighed every 2 days and water was added to return them to target weights.

Growth enhancer treatments were applied every two weeks throughout the experimental period. CaC_2_ (0.31 g per pot) was broadcast evenly on the soil surface to allow gradual Ca ion and acetylene gas release. GA_3_ (100 mg L^− 1^, 20 mL per pot) was applied as a soil drench around the plant base to ensure root zone availability. For the combined treatment (GE4), CaC_2_ (0.31 g) was first broadcast on the soil surface, followed by GA_3_ solution (100 mg L^− 1^, 20 mL) applied as a soil drench. GE1 and GE2 plants received 20 mL of distilled water as a drench to maintain constant soil moisture across treatments. On days when growth enhancer treatments coincided with irrigation event, the 20 mL liquid volume from the treatments was subtracted from the calculated irrigation requirement to maintain accurate field capacity levels. CaC_2_ dose and GA_3_ concentration were selected based on preliminary dose-response trials and prior reports of effective applications in crops under stress conditions [25, 28, 33]. All measurements were taken once during the final week before harvest at 85 DAT. This single time-point approach was chosen to assess the cumulative effects of the treatments on drought tolerance.

### Photosynthetic parameters

Photosynthetic parameters: net photosynthetic rate (A), stomatal conductance (g_sw_), intercellular CO_2_ concentration (Ci), and transpiration rate (E) were measured using a portable photosynthesis device (LI-COR 6400XT, LI-COR Incorporated, Lincoln, Nebraska, USA) fitted with an artificial light source (6400-02B LED). Measurements were recorded on the third fully expanded leaf from apex. Photosynthetic parameters were measured at ambient CO_2_ concentration (400 µmol mol^−1^), relative humidity (50–60%), photosynthetic photon flux density (1200 µmol m^−2^ s^−1^), and air flow rate (500 µmol s^−1^). Additionally, apparent mesophyll conductance (AMC = A/Ci), stomatal limit (Ls = 1 - Ci/Ca, where Ca is the ambient CO_2_ concentration), and nonstomatal limit (Lns = Ci/g_sw_) values were manually calculated [34].

### Pigment content

Four leaf disks were taken from the same leaf samples using a cork borer, placed in 20-mL amber glass vials, and immediately transported to the laboratory. Pigments were extracted from the leaf disks with 80% acetone. Samples were pipetted with 20 mL of 80% acetone and was stored in the refrigerator (4 °C) for 7 days. Subsequently, 1 mL of extract was aliquoted into a quartz cuvette and 1 mL of 80% acetone was pipetted into another cuvette to serve as blank. Absorbance was read at 646 nm and 663 nm in dim light in the cuvette port of a multiplate spectrometer (Multiskan GO, Thermo Scientific, Waltham, USA). Chlorophyll a (Chl a) and chlorophyll b (Chl b) contents were expressed as milligrams per gram fresh weight (mg g^− 1^ FW) in the following equations [35]:

$$chl\;a\left(mg\;g^{-1}\;FW\right)=\left(12.21A_{663}-2.81A_{646}\right)\times V\div\left(FW\times1000\right)$$

$$ch\;b\left(mg\;g^{-1}FW\right)=\left(20.13A_{646}-5.03A_{663}\right)\times V\div\left(FW\times1000\right)$$

Where V is final volume of extract in mL, and FW is total fresh weight of leaf disks in g. Chl a to Chl b ratios (Chl a/b = Chl a/Chl b) were then calculated.

### Electrolyte leakage

Electrolyte leakage (EL) was measured via conductivity [36] with modifications. Leaf disks were taken from the same leaf samples and immediately transported to the laboratory. To eliminate surface contamination, samples were washed with distilled water. Subsequently, 10 mL of distilled water were pipetted into each tube. The first set of samples were incubated at room temperature (24 °C) while orbitally shaken (100 rpm) for 24 h. The electrical conductivity reading of bathing solution (EC1) was measured using an EC meter (GLP 31, Crison Instruments, Spain). The second set of samples were autoclaved (120 °C) for 20 min. A second reading of the electrical conductivity (EC2) was measured after cooling the solution. EL index (%EL = EC1/EC2 × 100) was calculated.

### Proline

Proline (Pro) content was measured using the acidic ninhydrin assay [37]. Fresh leaf samples (the third from apex) were collected in the morning, put in ziplock bags, and immediately transported to the laboratory. Samples (0.5 g) were homogenized with 10 mL of 3% sulfosalicylic acid and then filtered. Subsequently 2 mL of samples were aliquoted and then mixed with 2 mL of acetic acid and 2 mL of acidic ninhydrin reagent. The mixtures were boiled for 1 h in a water bath, cool to room temperature, and pipetted with 4 mL of toluene. Absorbance of toluene top layer was read at 518 nm. Pro levels were expressed as micromoles per gram fresh weight (µmol g^− 1^ FW).

### Morpho-developmental traits

Plant height (PH) measured with a ruler, number of leaves (N_L_), canopy diameter (CD) measured with a measuring tape, and length of the central leaf vein were recorded at harvest (12 weeks after transplanting). The central leaf vein data were used to estimate total leaf area (LA) of the plants (cm^2^) based on the equation proposed by Campostrini and Yamanishi (2001) [38]. For shoot biomass determination, plants were carefully uprooted from the soil and washed free of soil particles using tap water, kept in hole punch plastic bags, labeled, and immediately transported to the laboratory. Shoots (leaves and stems) were separated from the roots, gently blotted dry with tissue paper and immediately weighed as shoot fresh weight (FWS).

### Statistical analysis

Data were analyzed as 3 × 4 factorial experiment (three water stress levels × four growth enhancer treatments) in a randomized complete block design with four biological replications using SAS^®^ version 9.4 PROC GLM. Where necessary, data were transformed using Box-Cox transformations to ensure normality of residuals. Two-way analysis of variance (ANOVA) was employed to test the fixed effects of water stress, growth enhancer, and their interaction [39]. Following the analytical method of Vargas et al. (2015) [40] for factorial experiments, when interaction effects were significant (*P* < 0.05), all treatment combination means were compared using Fisher’s protected least significant difference (LSD) test to detect synergistic effects and verify whether interactions were due to crossover (rank changes) or non-crossover (scale changes) patterns. If interactions were non-significant, only main effects were compared.

For multivariate analysis, individual replicate measurements from all treatment combinations (*n* = 48) across 15 measured traits (A, g_sw_, E, AMC, Ls, Lns, Chl a, Chl b, EL, Pro, PH, NL, LA, CD, FWS) were included. Raw data were first standardized using z-score transformation to zero mean and unit variance to remove scale differences among traits measured in different units. Subsequently, Pearson’s correlation coefficients were calculated for all pairwise trait combinations, assessed at three significance levels (∗∗∗*P* < 0.001; ∗∗*P* < 0.01; ∗*P* < 0.05). Principal component analysis (PCA) was performed on the standardized data using the Pearson correlation matrix, utilizing singular value decomposition to extract eigenvalues and eigenvectors. The first two principal components (Dim1 and Dim2) explaining the largest cumulative proportion of total variance were retained for biplot visualization. The PCA biplot shows both sample scores (treatment combinations as points) and variable loadings (trait vectors as arrows). Vector length indicates contribution to displayed variance and angles between vectors represent trait correlations. Graphs, correlation matrix, and PCA biplot were generated using OriginPro^®^ 2024b (OriginLab, Northampton, MA, USA).

## Results

### Photosynthetic parameters

**Fig. 1:**
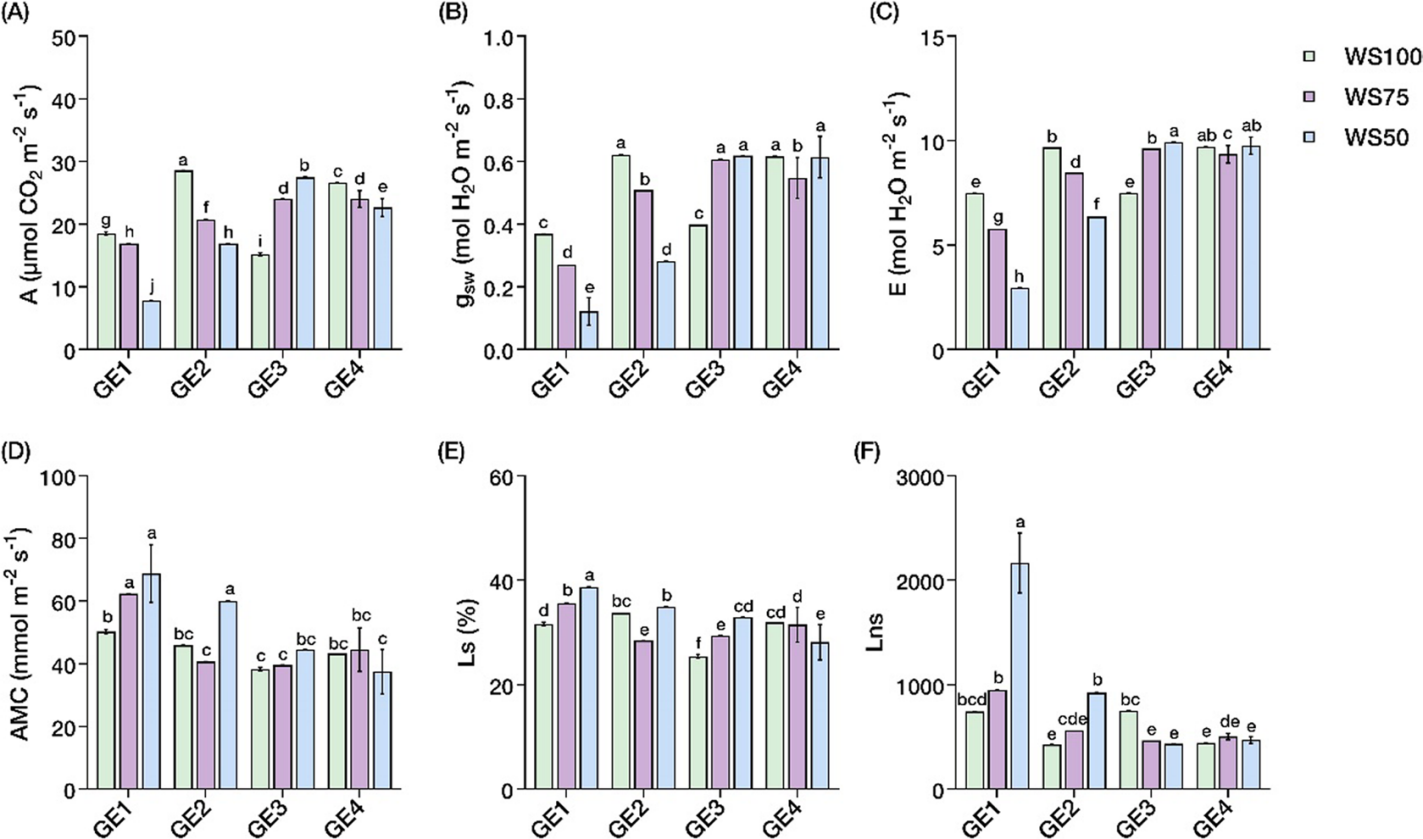
Changes in photosynthetic parameters in *Carica papaya* L. Interaction effects of water stress and growth enhancer treatments on **A** Net photosynthetic rate (A), **B** Stomatal conductance (g_sw_), **C** Transpiration rate (E), **D** Apparent mesophyll conductance (AMC), **E** Stomatal limit value (Ls), and **F** Nonstomatal limit value (LNS). Well-watered, 100% field capacity (WS100); mild water stress, 75% field capacity (WS75); severe water stress, 50% field capacity (WS50). Control, no growth enhancer (GE1); CaC_2_ (GE2); GA_3_ (GE3); CaC_2_ + GA_3_ (GE4) treatments. Mean ± SEs (*n* = 4) with different letter(s) above bars indicate significant differences according to least significant difference (LSD)

Water stress and growth enhancer treatments showed significant interaction effects (*P* < 0.05) on photosynthetic parameters (Suppl. Table S2). Under WS100, growth enhancers increased A than control (18.49 µmol CO_2_ m^− 2^ s^− 1^), with GE2 achieving highest rate (28.54 µmol CO_2_ m^− 2^ s^− 1^, + 54%), followed by GE4 (26.61 µmol CO_2_ m^− 2^ s^− 1^, + 44%). WS50 dramatically reduced A in control plants (7.78 µmol CO_2_ m^− 2^ s^− 1^, −58%), however, GA_3_-treated plants (WS50-GE3) maintained remarkably high A (27.48 µmol CO_2_ m^− 2^ s^− 1^), exceeding even WS100 controls by 49% (Fig. 1A). Meanwhile, g_sw_ and E followed similar patterns, with GE3 and GE4 treatments sustaining rates under water stress comparable to or exceeding well-watered controls (WS50-GE3 and WS50-GE4: ~0.61 mol H_2_O m^− 2^ s^− 1^ and ~ 9.56–9.77 mmol H_2_O m^− 2^ s^− 1^, each), while control plants showed 40–69% reductions (Fig. 1B, C).

Analysis of photosynthetic limitations revealed contrasting response patterns that distinguished growth enhancer effects. AMC showed high variability in control and CaC_2_-only treatments across water stress levels (59.91–68.77 mmol m^− 2^ s^− 1^ under WS50), whereas GA_3_-containing treatments (GE3 and GE4) demonstrated remarkable stability, maintaining AMC between 37.45 and 44.51 mmol m^− 2^ s^− 1^ regardless of water availability (Fig. 1D). Lns showed the most drastic treatment difference: WS50-GE1 showed catastrophically high values (2165.26) compared to all other treatments, while GE3 and GE4 consistently sustained Lns below 1000 across all water levels (Fig. 1F). Under WS50, Lns in growth enhancer treatments ranged from 434.11 to 922.92, indicating 80–95% reductions compared to controls. Ls increased gradually with water stress in control plants (31.6% to 38.7%) but remained lowest in GA_3_ treatments (WS100-GE3: 25.5%) (Fig. 1E).

### Pigment contents

**Fig. 2:**
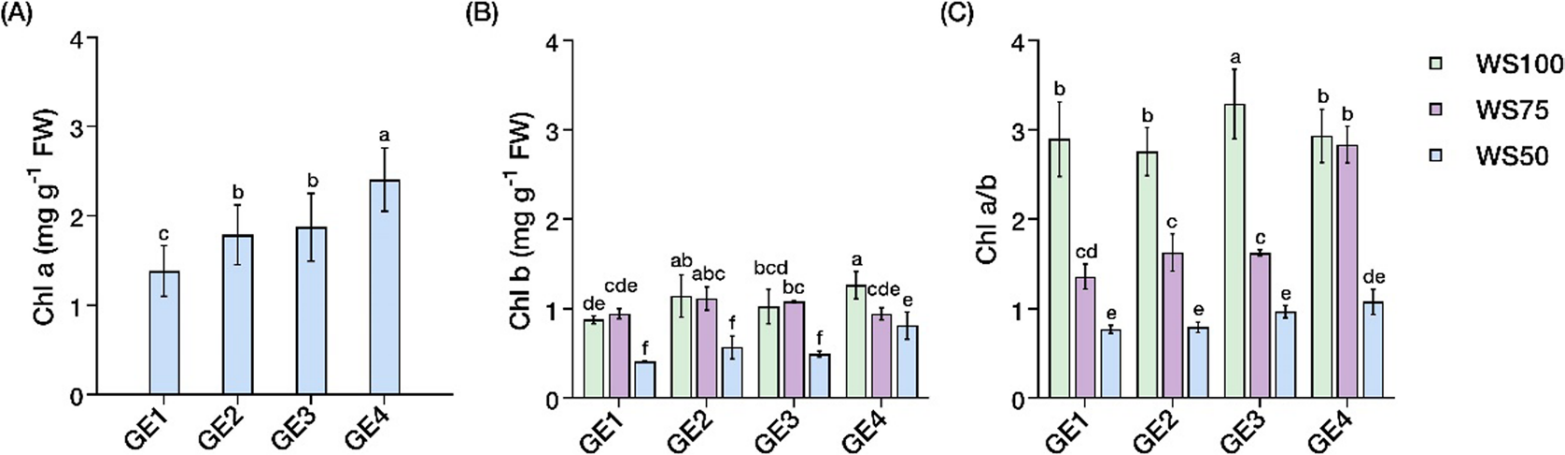
Changes in pigment contents in *Carica papaya* L. Independent effects of growth enhancer on **A** Chlorophyll a (Chl a). Interaction effects of water stress and growth enhancer treatments on **B** Chlorophyll b (Chl b) and **C** Chlorophyll a to chlorophyll b ratio (Chl a/b). Well-watered, 100% field capacity (WS100); mild water stress, 75% field capacity (WS75); severe water stress, 50% field capacity (WS50). Control, no growth enhancer (GE1); CaC_2_ (GE2); GA_3_ (GE3); CaC_2_ + GA_3_ (GE4) treatments. Mean ± SEs (*n* = 4) with different letter(s) above bars indicate significant differences according to least significant difference (LSD)

Water stress and growth enhancer treatments exhibited significant independent and interaction effects (*P* < 0.05) on pigment contents (Suppl. Table S2). Growth enhancer treatments influenced Chl a content independent of water stress, with GE4 producing the highest levels (2.41 mg g^− 1^ FW), indicating 75% increase over control (1.38 mg g^− 1^ FW). GE2 and GE3 achieved intermediate levels (~ 1.78–1.87 mg g^− 1^ FW) (Fig. 2A).Chl b showed interaction effects, remaining stable across growth enhancer treatments under WS100 and WS75 (0.88–1.27 mg g^− 1^ FW) but declining substantially under WS50 (0.41–0.81 mg g^− 1^ FW) across all treatments, indicating distinct sensitivity to severe water limitation (Fig. 2B).

Chl a/b showed water stress-dependent responses that varied with growth enhancer application. WS100 maintained relatively stable ratios (2.76–3.29) across treatments, while WS75 produced the most variable responses (1.36–2.84) depending on growth enhancer type. Under WS50, the ratios converged to intermediate values (0.77–1.08), reflecting varying sensitivity of Chl components to progressive water deficit (Fig. 2C).

### Stress markers

**Fig. 3:**
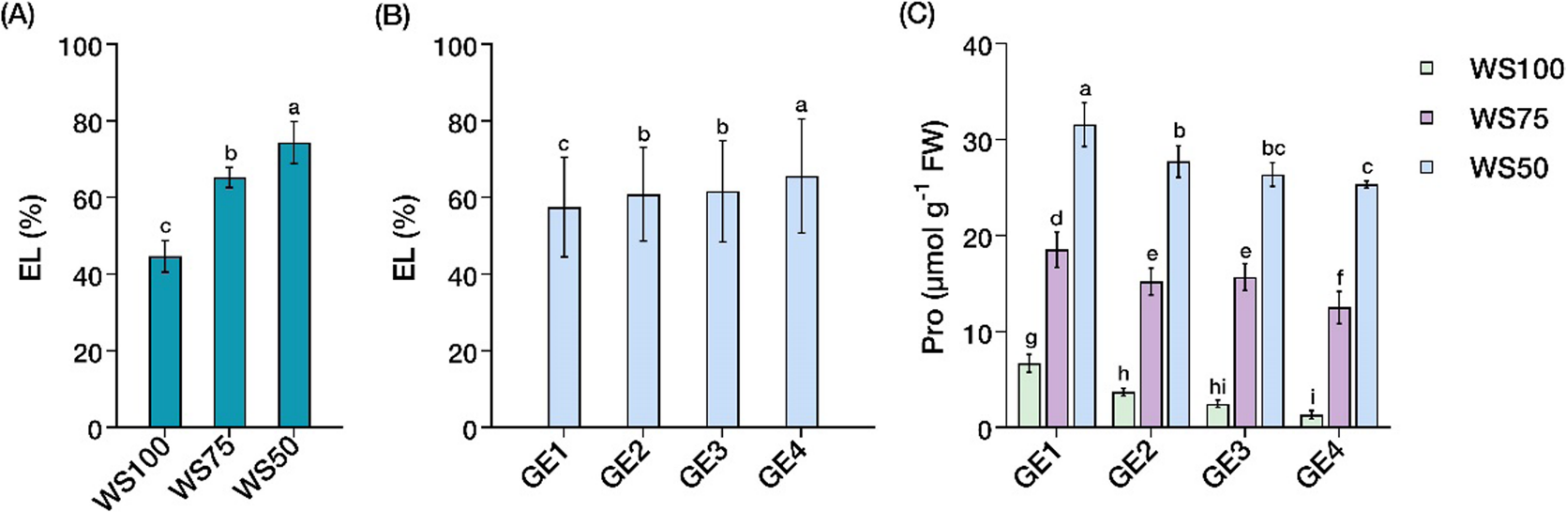
Changes in stress markers in *Carica papaya* L. Independent effects of water stress or growth enhancer on **A**,** B** Electrolyte leakage (EL). Interaction effects of water stress and growth enhancer treatments on **C** Proline content (Pro). Well-watered, 100% field capacity (WS100); mild water stress, 75% field capacity (WS75); severe water stress, 50% field capacity (WS50). Control, no growth enhancer (GE1); CaC_2_ (GE2); GA_3_ (GE3); CaC_2_ + GA_3_ (GE4) treatments. Mean ± SEs (*n* = 4) with different letter(s) above bars indicate significant differences according to least significant difference (LSD)

Water stress and growth enhancer treatments exhibited significant independent and interaction effects (*P* < 0.05) on stress markers (Suppl. Table S2). Water stress significantly increased EL in a dose-dependent manner: WS50 showed the highest membrane permeability (74%), followed by WS75 (65%) and WS100 (45%) (Fig. 3A). Growth enhancer treatments showed contrasting effects on membrane stability, with GE4 showing the highest EL (66%), followed by GE3 (62%) and GE2 (61%), while control recorded the lowest values (58%) (Fig. 3B). This pattern diverged from usual stress marker interpretations, where higher EL typically indicates greater damage.

Pro accumulation showed distinct interaction effects between water stress and growth enhancer treatment (Fig. 3C). Under well-watered conditions, control plants (WS100-GE1) accumulated the highest Pro content (6.7 µmol g^− 1^ FW), while growth enhancer treatments progressively reduced accumulation, with GE4 demonstrating the most pronounced reduction. This inverse relationship between Pro levels and growth enhancer application persisted across all water stress levels. Under WS50, Pro content increased substantially in all treatments compared to WS100, however, growth enhancer applications (particularly GE3 and GE4) consistently maintained lower Pro levels than controls at each corresponding water stress level, challenging conventional associations between Pro accumulation and stress tolerance.

### Morpho-developmental traits

**Fig. 4:**
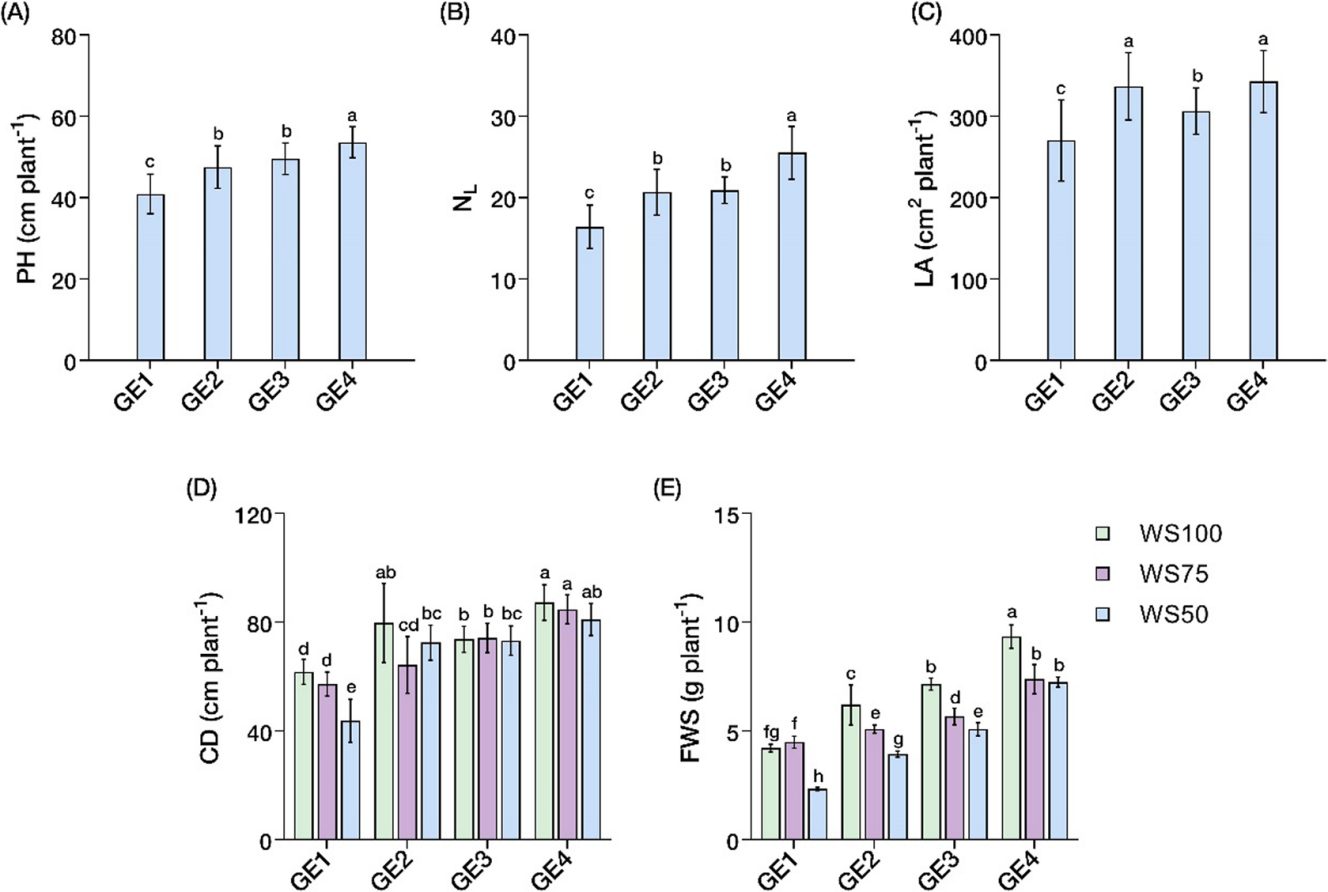
Changes in morpho-developmental traits of *Carica papaya* L. Independent effects of growth enhancer on **A** Plant height (PH) **B** Number of leaves (N_L_), and **C** Leaf area (LA). Interaction effects of water stress and growth enhancer treatments on **D** Canopy diameter (CD) and **E** Shoot fresh weight (FSW). Well-watered, 100% field capacity (WS100); mild water stress, 75% field capacity (WS75); severe water stress, 50% field capacity (WS50). Control, no growth enhancer (GE1); CaC_2_ (GE2); GA_3_ (GE3); CaC_2_ + GA_3_ (GE4) treatments. Mean ± SEs (*n* = 4) with different letter(s) above bars indicate significant differences according to least significant difference (LSD)

Water stress and growth enhancer treatments exhibited significant independent and interaction effects (*P* < 0.05) on morpho-developmental traits (Suppl. Table S2). Growth enhancer increased PH, N_L_, and LA independent of water stress effects. GE4 yielded the tallest plants (54 cm plant^− 1^) than control (40.9 cm plant^− 1^), with GE2 and GE3 yielding intermediate heights (~ 49 cm plant^− 1^) (Fig. 4A). Leaf development followed similar trends, with N_L_ increasing from 16 leaves in controls to 21–26 leaves per plant across growth enhancer treatments (Fig. 4B). LA was maximized under GE2 and GE4 treatments (~ 340 cm^2^ plant^− 1^, + 21% over control), while GE3 produced intermediate values (306 cm^2^ plant^− 1^) (Fig. 4C).

CD and FWS showed significant interaction effects, revealing differential responses to combined water stress and growth enhancer applications. GE4 treatment consistently maximized CD (~ 84.3 cm plant^−1^) across water stress levels, while severe drought minus growth enhancers (WS50-GE1) greatly reduced CD (43.8 cm plant^−1^, −29% vs. WS100-GE1) (Fig. 4D). The most dramatic treatment differentiation occurred in FWS: WS100-GE4 produced the highest biomass (9.34 g plant^−1^), while WS50-GE1 produced the lowest (2.34 g plant^−1^). Intriguingly, WS50-GE4 sustained high FWS (7.25 g plant^−1^, representing 78% of WS100-GE4), exceeding individual CaC_2_ or GA_3_ applications under optimal conditions and demonstrating synergistic drought mitigation (Fig. 4E).

### Correlations among measured traits

**Fig. 5:**
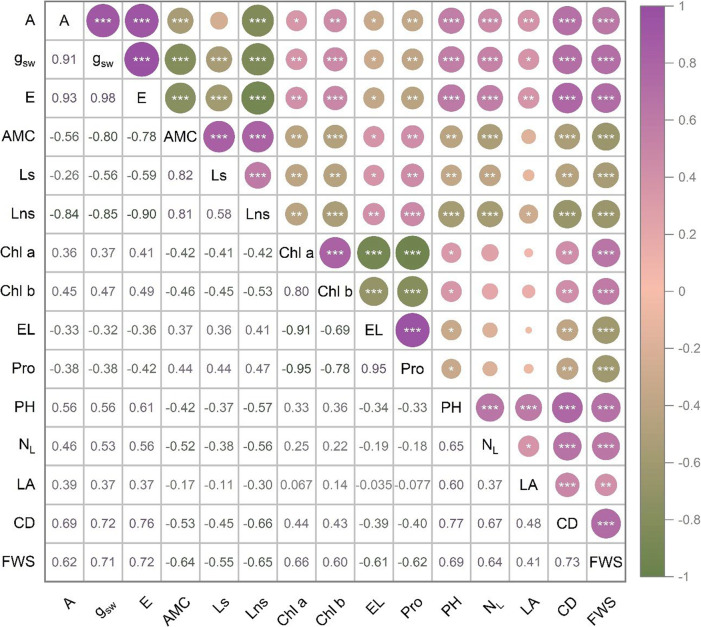
Correlation matrix showing the relationship among traits in *Carica papaya* L. under water stress and growth enhancer treatments. Photosynthetic parameters: net photosynthetic rate (A); stomatal conductance (g_sw_); transpiration rate (E); apparent mesophyll conductance (AMC); stomatal limit value (Ls), nonstomatal limit value (Lns), pigment contents: chlorophyll a (Chl a); chlorophyll b (Chl b), stress markers: electrolyte leakage (EL); proline (Pro), and morpho-developmental traits: plant height (PH); number of leaves (N_L_); leaf area (LA); canopy diameter (CD); shoot fresh weight (FSW). Data represents combined measurements from all treatment combinations (∗∗∗*P* < 0.001; ∗∗*P* < 0.01; ∗*P* < 0.05)

Correlation analysis unveiled distinct patterns of associations among the traits in *Carica papaya* L. subjected to water stress and growth enhancer treatments (Fig. 5). Photosynthetic parameters displayed remarkably strong positive correlations with each other, particularly between A and g_sw_ (*r* = 0.91), E (*r* = 0.98), and AMC (*r* = 0.93, all *P* < 0.001). These photosynthetic parameters showed consistently strong negative correlations with stress markers, most strikingly between A and EL (*r* = −0.91) and Pro (*r* = −0.95, all *P* < 0.001). Both Ls and Lns showed moderate to strong negative correlations with photosynthetic parameters excluding AMC, while showing positive associations with stress markers. Chl a and Chl b were positively correlated (*r* = 0.80, *P* < 0.001) and showed moderate positive associations with photosynthetic parameters while correlating negatively with stress markers. Morpho-developmental traits formed a highly interconnected cluster with strong positive correlations among themselves, primarily between CD and FSW (*r* = 0.73, *P* < 0.001), and positive correlations with A, g_sw_, and E while showing negative associations with stress markers. Intriguingly, morpho-developmental traits showed negative correlations with AMC, Ls, and Lns.

### Principal component analysis of measured traits

**Fig. 6:**
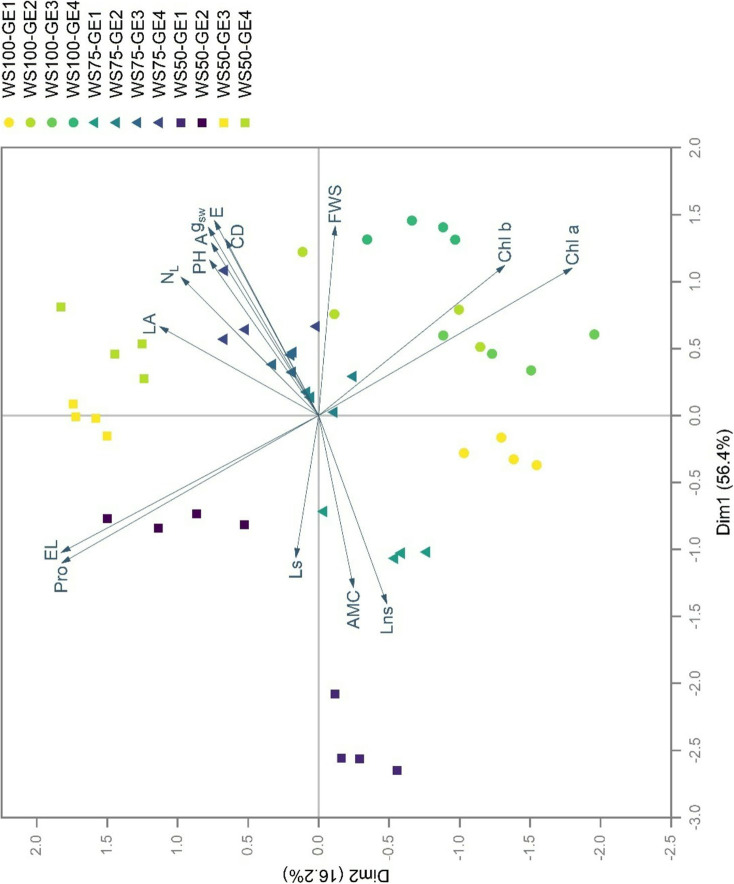
PCA biplot showing the relationship among measured traits in *Carica papaya* L. under water stress and growth enhancer treatments. Photosynthetic parameters: net photosynthetic rate (A); stomatal conductance (g_sw_); transpiration rate (E); apparent mesophyll conductance (AMC); stomatal limit value (Ls), nonstomatal limit value (Lns), pigment contents: chlorophyll a (Chl a); chlorophyll b (Chl b), stress markers: electrolyte leakage (EL); proline (Pro), and morpho-developmental traits: plant height (PH); number of leaves (N_L_); leaf area (LA); canopy diameter (CD); shoot fresh weight (FSW). Well-watered, 100% field capacity (WS100); mild water stress, 75% field capacity (WS75); severe water stress, 50% field capacity (WS50). Control, no growth enhancer (GE1); CaC_2_ (GE2); GA_3_ (GE3); CaC_2_ + GA_3_ (GE4) treatments

PCA revealed distinct clustering patterns among water stress and growth enhancer treatments, with the first two dimensions explaining 74.6% of the total variance (Dim1: 56.4%, Dim2: 18.2%) (Fig. 6). The PCA biplot showed clear separation of treatments along both axes, with WS50-GE1 treatment clustering in the extreme negative region of Dim1 and exhibiting strong associations with stress markers (EL, Pro). These stress marker vectors showed a perpendicular orientation to performance traits (morpho-developmental and photosynthetic) with Pro extending along Dim2 while performance traits aligned with Dim1. This orthogonal relationship was most dramatically shown by the WS50-GE1 treatment, which clustered in the extreme negative region of Dim1 while associating with stress marker vectors in the upper-left quadrant. This implies the combination of highest stress marker accumulation with poorest performance.

Growth enhancer-treated plants under severe drought (WS50-GE2, WS50-GE3, and WS50-GE4) positioned intermediately between stress markers and morpho-developmental traits, indicating partial uncoupling of stress responses from performance decline. Both WS100-GE3 and WS100-GE4 samples were dispersed in the positive region of Dim1, oriented in the direction of morpho-developmental traits (PH, LA, N_L_, CD, FSW). However, they are positioned at a slight distance from these trait vectors. Photosynthetic parameters showed contrasting orientations, with A, g_sw_, and E vectors pointing toward the upper right quadrant, while AMC, Ls, and Lns displayed opposing vectors directed toward the left quadrants. WS75 treatments displayed intermediate positioning, with WS75-GE2 and WS75-GE3 samples clustering near photosynthetic parameters (A, g_sw_, E) in the upper quadrants. WS100-GE2 showed associations with pigment composition (Chl a, Chl b), with the vectors extending toward the lower right quadrant where these samples dominated. AMC and Lns loading vectors aligned with WS75-GE1 samples in the left quadrants, indicating distinct physiological adjustments under mild water stress without growth enhancers.

## Discussion

Our investigation provides new insights into drought stress mitigation strategies for tropical fruit crops, indicating that CaC_2_ and GA_3_ co-application adjusts the physiological responses of papaya to water stress. The most notable discovery focuses on the paradoxical uncoupling of traditional stress markers from plant performance, a trend that challenges conventional drought physiology paradigms and suggests activation of alternative metabolic pathways that bypass classical stress response mechanisms.

The preservation of photosynthetic capacity (27.48 µmol CO_2_ m^−2^ s^−1^) under severe water stress by GA_3_-treated plants (WS50-GE3) indicates a remarkable deviation from typical drought responses. This result contrasts with established trends in other tropical species, where photosynthetic rates typically reduce by 60–80% under similar stress conditions [41–43]. The extraordinarily high correlation between photosynthetic parameters (A and g_sw_: *r* = 0.91, A and E: *r* = 0.98) shows the integrated nature of gas exchange processes, yet growth enhancer treatments fundamentally adjusted these relationships. Our finding supports previous findings in drought-stressed faba beans and canola, where exogenous GA_3_ application resulted in elevated photosynthesis rates [33, 44]. Remarkably, the magnitude of photosynthetic parameters preservation in papaya exceeds prior reports which suggests species-specific optimization of GA_3_ mediated stress tolerance responses, though the exact mechanisms require further investigation.

The differential response between Ls and Lns offers mechanistic insights into this phenomenon. While control plants showed catastrophic Lns (2165.26) under severe water stress conditions, GA_3_ and combined treatments (WS50-GE3 and WS50-GE4) maintained values below 1000, indicating preservation of mesophyll conductance and related biochemical processes. This finding parallels reports in soybean and heat-stressed wheat, where GA_3_ treatment protected Rubisco activity [45, 46]. The stability of AMC across water regimes in GA_3_-treated plants as well as combined treatments further confirms the protection of cellular CO_2_ diffusion pathways, a trait previously documented in resurrection plants and mesophytes [47, 48].

A major finding is the orthogonal relationship between Pro contents and plant performance traits revealed by PCA. This pattern shows that Pro accumulation indicates a stress response that works independently of growth maintenance mechanisms, consistent with its role as a stress indicator rather than a direct performance enhancer. Conventional understanding ranks Pro as an osmolyte with accumulation levels positively correlating with stress tolerance [49, 50]. Yet, our results show that Pro accumulation is a distinct stress response axis, independent of growth and photosynthetic parameters. This orthogonality is most strikingly displayed by the WS50-GE1 treatment, which clusters tightly with the Pro vector in the upper-left quadrant while exhibiting the poorest performance traits. In contrast, growth enhancer-treated plants subjected to the same severe drought conditions (WS50-GE3 and WS50-GE4) positioned away from the Pro vector while keeping substantially better performance. This pattern shows that Pro accumulation may serve as a metabolic “emergency brake” rather than performance enhancer, consistent with earlier work on drought-tolerant wheats where superior genotypes reduced Pro levels compared to sensitive varieties [51].

Our findings suggest CaC_2_ and GA_3_ co-application may promote alternative osmotic adjustment mechanisms beyond the Pro pathway. These could include enhanced synthesis of soluble sugars (such as trehalose and other carbohydrates), glycine betaine, or polyamines [52–54], though direct measurement of these compounds would be needed to confirm this hypothesis. The slightly elevated EL in growth enhancer treatments (GE4: 66% vs. GE1: 58%) despite better performance, suggests controlled membrane permeabilization that may accelerate osmotic adjustment without inducing the Pro-mediated stress response axis [55]. This hypothesis aligns with emerging models of priming-induced stress memory, whereby pre-exposure to mild stressors enhances subsequent stress responses through epigenetic modifications [56].

The superior performance of CaC_2_ treatment, primarily in sustaining photosynthesis under well-watered conditions (WS100: 54% increase), establishes the roles of Ca in structural integrity and stress signaling. During dehydration, Ca is a cell wall stabilizer, cross-linking pectin molecules to maintain cellular architecture as turgor pressure declines [57, 58]. Our PCA space displaying WS100-GE2 samples associating with the chlorophyll vectors suggests that Ca fortification of cell walls may preserve chloroplast positioning and light-harvesting efficiency. The sustained photosynthesis under drought (27.48 µmol CO_2_ m^-2^ s^-1^ in WS50-GE3) likely reflect the role of Ca in preventing cell wall collapse that often disrupt mesophyll CO_2_ diffusion pathways [59]. Beyond structural support, the release of Ca ions and acetylene gas by CaC_2_ creates a unique biochemical environment [28, 60]. The conversion of acetylene to ethylene via microbial activity in the soil [25, 61] establishes a sustained, low-level ethylene exposure that likely cooperates synergistically with GA_3_ signaling pathways.

While direct evidence for ethylene-GA_3_ synergism in drought responses remains scarce, extensive research has documented complex interactions between these hormones in various physiological processes. Ethylene and gibberellins exhibit antagonistic and synergistic interactions depending on plant development and stress conditions [62, 63]. In Arabidopsis and tomato, adding ethylene can strongly enhance or prevent the effects of gibberellins [64, 65]. GA_3_ treatment likely promotes DELLA protein degradation through the GID1 receptor pathway [66], potentially releasing growth limits that are typically maintained under drought stress. The Ca component further amplifies stress responses through its role as a secondary messenger, activating Ca-dependent protein kinases (CDPKs) that phosphorylate key transcription factors involved in stress-responsive gene expression [21, 67]. These converging signals: Ca, prospective ethylene, and GA_3_, may rewire the stress response network to prioritize growth conservation over defensive metabolites generation, as evidenced by reduced Pro levels in treated plants. However, further molecular validation would be needed to confirm the proposed signaling interactions.

GE4 treatment induced a 75% increase in Chl a content, signifying the powerful synergistic effects of CaC_2_ and GA_3_ application on pigment composition. The strong positive correlation between Chl a and Chl b (*r* = 0.80) indicates coordinated regulation of light-harvesting complex biosynthesis, while the differential responses of Chl a/b ratios under varying water levels suggest modifications in photosynthetic apparatus organization. Similar chlorophyll increments have been recorded in both GA_3_-treated canola and maize under salinity stress [68, 69], though direct comparisons across species and stress conditions remain difficult. The preservation of chlorophyll content under severe water stress by growth enhancer treatments likely involves multiple defense mechanisms, with GA_3_ upregulating chlorophyll biosynthesis while suppressing chlorophyllase activity [70] and Ca stabilizing thylakoid membrane structure and protecting photosystem II from photoinhibition [71]. The synergistic action of these compounds in our GE4 treatment may initiate an optimal condition for photosynthetic apparatus protection, explaining the excellent chlorophyll preservation observed even under severe water limitations.

The enhanced morpho-developmental traits under GE4 treatment, particularly the maintenance of FSW under severe water stress (7.25 g plant^−1^ vs. 2.34 g plant^−1^ in controls), reveals sophisticated resource allocation strategies. The strong positive correlations among the traits (CD and FSW: *r* = 0.73) reveal coordinated growth responses, while their moderate to strong negative correlations with AMC, Ls, and Lns suggest that growth enhancement occurs via mechanisms independent of mesophyll-level photosynthetic adjustments. PCA analysis elegantly captures this phenomenon, with GE4 plants under optimal conditions (WS100-GE4) clustering with morpho-developmental trait vectors while maintaining proximity to photosynthetic parameters in the positive region of Dim1. This unique positioning in the PCA space, accounting for 56.4% variance, suggests that GE4 treatment generates a unique physiological state that transcends classical growth-defense trade-offs. The clear separation along Dim1 between stressed controls and growth enhancer treatments under severe stress (WS50-GE3 and WS50-GE4) indicates partial decoupling of stress perception from growth inhibition, reinforcing the potential for hormone-mediated breach of evolutionary constraints similar to CRISPR-edited rice lines with modified GA metabolism [72].

The correlation matrix reveals a complex network of physiological interactions that reshapes our knowledge of stress responses. The consistent negative correlations between all photosynthetic parameters and stress markers are significant. This enhanced coupling suggests growth enhancer treatments amplify the sensitivity of physiological networks, enabling responsive and integrated stress management systems. However, the orthogonal relationship between Pro and performance traits revealed by PCA indicates that not all stress responses are coupled. Particularly remarkable is the intermediate positioning of WS75 treatments in the multivariate space, with WS75-GE2 and WS75-GE3 samples clustering nearby photosynthetic parameter vectors in the upper quadrants. This positioning, combined with their proximity to the intersection of Dim1 and Dim2, shows that mild water stress combined with growth enhancers may represent an optimal physiological state, where stress priming enhances performance without triggering costly defensive responses.

Our findings carry profound implications for developing climate-adaptive cultivation strategies in tropical fruit production systems. The ability of combined CaC_2_ and GA_3_ applications to sustain 78% of optimal shoot biomass under severe drought demonstrates promising potential for stress mitigation that needs further validation under field conditions. PCA shows that this performance enhancement creates a fundamentally different plant physiological state, as evidenced by 74.6% variance explained by two principal components. Moreover, the orthogonal relationship between Pro accumulation and performance traits largely suggests that traditional stress markers may not accurately predict plant performance under hormone-mediated stress mitigation, necessitating a reevaluation of drought tolerance screening protocols. The relatively low cost and availability of CaC_2_ and GA_3_ make this technology particularly suitable for smallholder farmers in developing tropical regions. The slow-release nature of CaC_2_ also reduces application frequency, addressing labor constraints common in tropical agriculture.

**Fig. 7:**
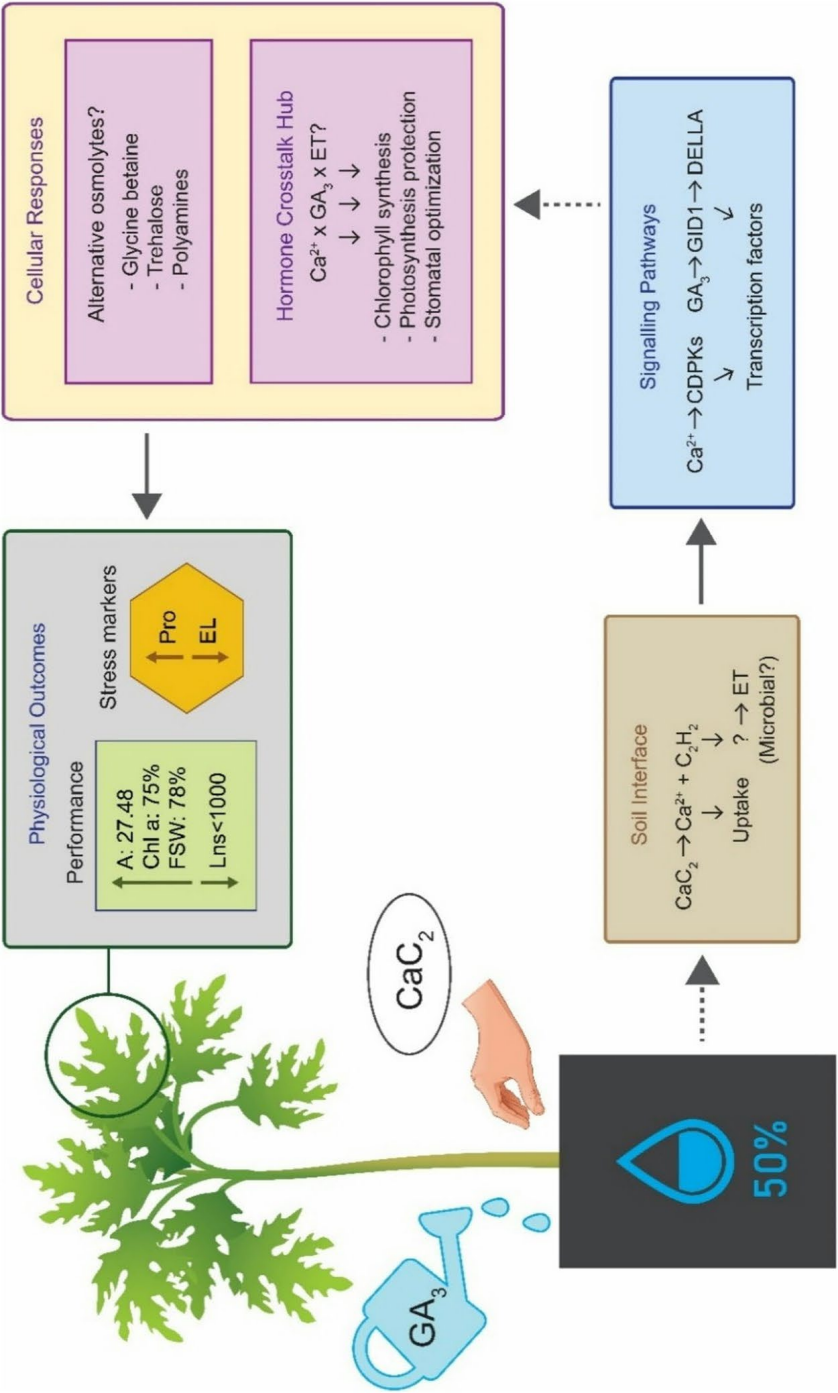
Proposed mechanistic model of calcium carbide (CaC_2_) and gibberellic acid (GA_3_) synergistic action in enhancing drought resilience in *Carica papaya* L. under severe water stress (50% field capacity). Solid arrows indicate established pathways; dashed arrows indicate proposed mechanisms requiring further validation. Performance-related traits: net photosynthetic rate (A); chlorophyll a (Chl a); shoot fresh weight (FSW); nonstomatal limit value (Lns), stress markers: proline (Pro); electrolyte leakage (EL), ethylene (ET)

Based on our comprehensive analyses, we propose a mechanistic model explaining how CaC_2_ and GA_3_ co-application enhances drought resilience in papaya (Fig. 7). Under severe water stress, CaC_2_ releases Ca ions that activate CDPKs while producing acetylene potentially converted to ethylene via soil microbes. These signals converge with GA_3_-mediated DELLA protein degradation via the GID1 receptor pathway, creating a hormone crosstalk hub that rewires stress response networks. Our data supports this model via maintenance of photosynthetic capacity despite severe drought, increase in Chl a, dramatic reduction in Lns, and orthogonal relationship between stress markers and performance traits uncovered by PCA. This hormone-mediated rewiring appears to activate alternative osmotic adjustment pathways beyond Pro accumulation This framework establishes how strategic hormone combinations can break evolutionary trade-offs between stress tolerance and productivity, offering directly deployable solutions for climate-resilient agriculture.

Future investigation should focus on elucidating the precise mechanisms underlying the observed responses. Direct measurement of ethylene release from CaC_2_ under our experimental conditions, alternative osmolytes quantification, and transcriptomic analysis of hormone-responsive genes would provide deeper mechanistic insights. Field trials under natural drought conditions would further confirm the practical applicability of this approach for commercial papaya production.

## Conclusion

This study uncovers that combined calcium carbide and gibberellic acid modify drought response mechanism in papaya, facilitating maintenance of growth and photosynthesis under water stress. The orthogonal relationship between proline levels and performance traits suggests that growth enhancer treatments activate stress tolerance pathways that diverge from conventional osmotic adjustment mechanisms. Strikingly, the inverse relationship between traditional stress markers and plant performance traits implies that cellular stress perception can be decoupled from growth suppression via targeted hormonal mediation. The biochemical milieu created by the dual release of calcium ions and potential ethylene from calcium carbide, when synchronized with GA_3_ signals, appears to prime a homeostatic state where plants maintain growth despite environmental cues that would typically trigger conservative survival strategies. This hormone-mediated breaking of evolutionary trade-offs offers directly deployable solutions for tropical agriculture facing climate change. Beyond field applications, our findings reveal new insights where advantageous hormone interactions may be permanently encoded into crop genomes, changing how we engineer stress tolerance for tomorrow’s agriculture.

## Supplementary Information

Supplementary Material 1.

Supplementary Material 2.

## Data Availability

The data and materials supporting this study’s findings are available from the corresponding author upon request.

## Abbreviations

WS100: Well-watered, 100% field capacity
WS75: Mild water stress, 75% field capacity
WS50: Severe water stress, 50% field capacity
GE1: Control, no growth enhancer
GE2: CaC_2_ treatment
GE3: GA_3_ treatment
GE4: CaC_2_ + GA_3_ combined treatment
CaC_2_: Calcium carbide
GA_3_: Gibberellic acid
Ca: Calcium
Ca^2+^: Calcium ions
ET: Ethylene
A: Net photosynthetic rate
g_sw_: Stomatal conductance
E: Transpiration rate
Ci: Intercellular CO_2_ concentration
Ca: Ambient CO_2_ concentration
AMC: Apparent mesophyll conductance
Ls: Stomatal limit
Lns: Nonstomatal limit
Chl a: Chlorophyll a
Chl b: Chlorophyll b
Chl a/b: Chlorophyll a to chlorophyll b ratio
EL: Electrolyte leakage
Pro: Proline
PH: Plant height
N_L_: Number of leaves
LA: Leaf area
CD: Canopy diameter
FWS: Shoot fresh weight
FW: Fresh weight
PCA: Principal component analysis
LSD: Least significant difference
ANOVA: Analysis of variance
CDPKs: Calcium-dependent protein kinases
DELLA: DELLA proteins (growth repressors)
GID1: Gibberellin insensitive dwarf 1 (receptor)
PGR: Plant growth regulators
DAT: Days after transplanting
