# Supplementary material for: Calcium carbide and gibberellic acid co-application enhances drought resilience in papaya (Carica papaya L.) by modulating photosynthetic efficiency and stress markers
Source: BMC Plant Biol. 2026 Jan 16;26:281. doi: 10.1186/s12870-025-07845-4 (PMC12895614; doi:10.1186/s12870-025-07845-4)
Supplement: Supplementary file 1 — Supplementary Material 1. [file 12870_2025_7845_MOESM1_ESM.docx]

**Suppl. Table S2** Results of two-way ANOVA test and level of significance for photosynthetic parameters: net photosynthetic rate (A); stomatal conductance (g_sw_); transpiration rate (E); apparent mesophyll conductance (AMC); stomatal limit values (Ls), nonstomatal limit value (Lns), pigment contents: chlorophyll a (Chl a); chlorophyll b (Chl b), stress markers: electrolyte leakage (EL); proline (Pro), and morpho-developmental traits: plant height (PH); number of leaves (N_L_); leaf area (LA); canopy diameter (CD); shoot fresh weight (FSW). Data represents combined measurements from all treatment combinations (∗∗∗P < 0.001; ∗∗P < 0.01; ∗P < 0.05).

| Parameters | WS | GE | WS x GE |
| --- | --- | --- | --- |
| A | 117.93*** | 587.67*** | 366.70*** |
| g_sw_ | 22.37*** | 236.57*** | 68.70*** |
| E | 38.79*** | 572.67*** | 159.53*** |
| Ls | 21.96*** | 45.58*** | 23.84*** |
| Lns | 59.83*** | 551.69*** | 198.81*** |
| Chl a | 686.03*** | 47.70*** | 1.67^NS^ |
| Chl b | 74.89*** | 9.35*** | 3.37* |
| EL | 365.81*** | 13.06*** | 1.18^NS^ |
| Pro | 2098.26*** | 73.70*** | 4.52** |
| PH | 2.76^NS^ | 22.30*** | 1.65^NS^ |
| N_L_ | 0.08^NS^ | 22.60*** | 1.07^NS^ |
| LA | 0.48^NS^ | 11.51*** | 1.04^NS^ |
| CD | 6.19** | 43.18*** | 2.80* |
| FWS | 229.94*** | 524.02*** | 15.25*** |
