# Supplementary material for: Calcium carbide and gibberellic acid co-application enhances drought resilience in papaya (Carica papaya L.) by modulating photosynthetic efficiency and stress markers
Source: BMC Plant Biol. 2026 Jan 16;26:281. doi: 10.1186/s12870-025-07845-4 (PMC12895614; doi:10.1186/s12870-025-07845-4)
Supplement: Supplementary file 2 — Supplementary Material 2. [file 12870_2025_7845_MOESM2_ESM.docx]

**Suppl. Table S1** Selected physicochemical properties of "Munchong" soil series.

| Properties | Value | |  |
| --- | --- | --- | --- |
| Soil texture | | Clay | |
| Sand (%) | | 62.70 | |
| Silt (%) | | 10.89 | |
| Clay (%) | | 26.21 | |
| Bulk density (g cm^-3^) | | 0.79 | |
| Porosity (%) | | 70.59 | |
| pH (H_2_O) | | 4.68 | |
| Cation exchange capacity | | 8.00 | |
| Total carbon (C) (%) | | 1.60 | |
| Total nitrogen (N) (%) | | 0.20 | |
